# Management of a 25-Year-Old Female Patient With Limb-Girdle Muscular Dystrophy With Physiotherapy: A Case Report

**DOI:** 10.7759/cureus.51428

**Published:** 2024-01-01

**Authors:** Sojwal P Nandanwar, Swadha P Udhoji, Raghumahanti Raghuveer

**Affiliations:** 1 Neurophysiotherapy, Ravi Nair Physiotherapy College, Datta Meghe Institute of Higher Education & Research, Wardha, IND; 2 Neurophysiotherapy, Ravi Nair Physiotherapy College, Datta Meghe Institute of Higher Education and Research, Wardha, IND

**Keywords:** limb-girdle muscle dystrophy, dynamic balance and postural stability, gait, range of motion (rom), muscle strength, fatigue, neurophysiotherapy, proximal muscle weakness, limb-girdle muscular dystrophy type2a (lgmd2a)

## Abstract

Limb-girdle muscular dystrophy (LGMD) is a collection of neuromuscular diseases that develop gradually and are rare, genetically, and clinically diverse. The weakness in muscles affecting the shoulder and pelvic girdles is a defining feature of LGMD. Calpainopathy is another name for limb-girdle muscular dystrophy type 2A (LGMD2A). Limb-girdle muscular dystrophy type 2A results from alterations in the calpain-3 (CAPN3) gene, which results in a CAPN3 protein shortage. Gower's sign is most commonly found in LGMD2A. The prevalence ranges from one person in every 14,500 to one in every 123,000. We present a case of a 25-year-old hypotensive female patient who complained of weakness in all four limbs and easy fatigue with a positive Gower’s sign. For subsequent management, the neurologist referred the patient to the physical therapy department. The physical therapy goals included enhanced muscle strength, increased joint mobility, reduced fatigue, normalizing gait, and building dynamic balance and postural stability. Diagnosing LGMD clinical variability is important, emphasizing the importance of precise subtype identification and tailoring therapy. Tackling specific muscular deficits and functional restrictions emerges as a critical component in the holistic care of LGMD by physiotherapists. Continuous monitoring and evaluation using appropriate scales and measurements are essential for tracking performance and tailoring treatment strategies. Regular follow-up consultations with the physiotherapist are needed to identify changes in an individual's health and alter the treatment plan accordingly.

## Introduction

Limb-girdle muscular dystrophy (LGMD) is a term that refers to a diverse collection of muscle illnesses defined by a proximal occurrence of limb-girdle weakness. A set of progressive, uncommon, hereditary, and medically various neuromuscular diseases is known as LGMD [1, 2]. It is a group of hereditary conditions that cause progressive skeletal muscle degradation, leading to muscular weakness and physical impairment [3]. Limb-girdle muscular dystrophy type 2A (LGMD2A) is one of the most frequent adult-onset muscular dystrophies presenting with limb-girdle weakness, and it is the fourth most common genetic cause of muscle weakness. Calpaniopathy is another term for LGMD2A. Calpainopathy (autosomal recessive variety) is characterized by symmetric and increasing weakening of the proximal limb-girdle muscles [4]. Although both are prevalent, LGMD autosomal recessive variants are more prevalent than autosomal dominant variants.

Limb-girdle muscular dystrophy is characterized by genetic defects that result in the breakdown of particular proteins required for skeletal muscle growth and function. In an autosomal recessive inheritance, calpain-3 (CAPN3) alleles must be defective. A lack of the CAPN3 protein, which is necessary for muscle cell function, results from a mutation in the calpain-3 gene. A lack of CAPN3 results in abnormal proteins accumulating in muscle cells, which leads to progressive muscular atrophy and weakness [5]. The global frequency of LGMD is estimated to be 1one in 14,500-45,000 [6]. Calpainopathy is prevalent in all autosomal dominant and recessive forms of LGMD, ranging from 40% to 50% in Turkey, Bulgaria, and Indian populations [7].

Symmetrical muscle atrophy and weakness are hallmarks of this type of LGMD, especially in the shoulder and pelvic girdle muscles. People with LGMD2A may exhibit Gower's sign, defined as having trouble standing up from a sitting or reclining position without the aid of hands because the disease gradually weakens the muscles and impairs their ability to function. Patients with LGMD have increasing weakness in the muscles of the shoulder and pelvic girdle, resulting in handicaps ranging from little difficulty walking to severe incapacity. Understanding whether additional clinical factors are associated with this dysfunction and LGMD subtypes related to an increased risk of developing cardiac and respiratory problems could facilitate the clinician's ability to provide an accurate forecast and arrange follow-up therapy [8, 9].

Gower's sign, which is commonly present in patients with calaniopathy and a clinical symptom in persons with particular neuromuscular disorders, is most frequently observed in LGMD2A [9]. When the patient experiences cardiac and respiratory issues, it is advised that they breathe quickly and deeply. Patients could suffer from dyspnea during rest or physical activity, depending on the severity of muscle weakness. This sign of diaphragmatic weakness, which can be a defining feature of LGMD2A, is present [5]. By helping patients maintain their general physical function and their muscular strength and flexibility, physiotherapy plays a significant role in controlling LGMD [10]. Improved functional capacity for everyday duties, increased muscle strength, and improved cardiovascular health are all goals of treating calpaniopathy. Even those with chronic diseases can benefit from exercise in terms of mood and mental health. Overtraining or prolonged exercise (high-intensity training) may be harmful, raising the risk of accidents and falls due to muscle weakness and potentially causing damage to the muscles. Exercise programs should be tailored to each individual and carried out under supervision to avoid fatigue and hasten the condition's slow progression [11-14].

Exercises with a low-to-moderate intensity, like range-of-motion drills, stationary cycling, and aqua therapy, can help maintain muscle function, increase joint mobility, and enhance overall physical well-being. Strenuous or demanding exercises may not be suitable for those with LGMD since they could weaken them and harm their muscles. When considering an exercise program for a person with calpaniopathy, it is imperative to see a medical expert with experience treating patients with muscular dystrophy [14].

## Case presentation

A 25-year-old female hypotensive patient came to the outpatient department with complaints of symmetrical weakness in the proximal muscle of both upper and lower limbs for five years with slow progression. She had difficulty ascending the staircase, jogging, rising from sitting on the floor, and extending her arms above her head. There is no history of twitching, limb discomfort, cranial nerve involvement, or sensory complaints, and no bladder or bowel involvement. There was a history of breathlessness when performing an activity. The patient had contracted COVID-19 in 2019 and a surgical history of appendicitis in 2017. There was no history of addictive substances or drug use. There were no abnormal findings in the birth history or delays in developmental milestones. According to her family history, her 40-year-old sister had been in a wheelchair for the past 20 years due to a similar limb weakness.

Clinical findings

The patient was afebrile on general examination, with a heart rate of 70 beats/min, a blood pressure of 110/70 mm Hg, and a respiratory rate of 13 breaths/min. Cardiorespiratory system evaluation was routine. Her inspiratory and expiratory ratios were 2:1. Cognitive function, speech, and cranial nerves were intact after a neurological test. Her sensory system was intact. She presented with weakness in both shoulders, wasting in both deltoids, pseudo-hypertrophy in both calves and normalized tone in all four limbs. Weakness was present in the fingers and wrist. Table 1 presents the results of manual muscle testing (MMT), which was a strength measure for the patient.

**Table 1:** Strength ratings (manual muscle testing) by Medical Research Council grading 2: complete range of motion with gravity eliminated; 3: complete range of motion in opposition to gravity; 4: complete range of motion with minimum resistance against gravity; 5: complete range of motion with maximum resistance against gravity The ability to perform test movements in a position with reduced gravity is rated at 2 for trunk power. The ability to perform test movement or maintain test position under gravity and under pressure that is slightly less than moderate (slight to moderate) for trunk power is rated 4(+ &-). The capacity to finish the test movement and maintain the test position while applying the maximum amount of force for trunk power is rated 5.

Muscle	Pre-rehabilitation on the left side	Pre-rehabilitation on the right side	Post-rehabilitation on the left side	Post-rehabilitation on the right side
Shoulder flexors	3/5	3/5	4/5	4/5
Shoulder extensors	3/5	3/5	4/5	4/5
Shoulder abductors	3/5	3/5	4/5	4/5
Shoulder adductors	3/5	3/5	4/5	4/5
Elbow flexors	4/5	4/5	5/5	5/5
Elbow extensors	4/5	4/5	5/5	5/5
Wrist flexors	4/5	4/5	5/5	5/5
Wrist extensors	3/5	3/5	4/5	4/5
Hip flexors	3/5	3/5	4/5	4/5
Hip extensors	3/5	3/5	4/5	4/5
Hip abductors	2/5	2/5	3/5	3/5
Hip adductors	2/5	2/5	3/5	3/5
Knee flexors	4/5	4/5	5/5	5/5
Knee extensors	4/5	4/5	5/5	5/5
Ankle dorsiflexors	3/5	3/5	4/5	4/5
Ankle plantar flexors	3/5	3/5	4/5	4/5
Trunk flexors	2/5	2/5	3/5	3/5
Trunk extensors	2/5	2/5	3/5	3/5
Trunk rotators	2/5	2/5	4/5	4/5

Gower's signs were positive; all deep tendon reflexes and superficial reflexes were intact, and the plantar responses were flexors bilateral. Her gait was waddling. The respiratory function was reduced and noticeable, and the gastrointestinal system and cardiovascular function were unremarkable. On respiratory examination, air entry was bilaterally reduced. On assessment, chest expansion at the level of the axillary, nipple, and xiphoid processes revealed differences of 2 cm, 2 cm, and 2 cm, respectively. The electrocardiogram, chest radiography, echocardiogram, and ultrasonography of the abdomen were all normal. Nerve conduction tests were normal. Muscular dystrophy was discovered through a muscle biopsy. Cost constraints prevented DNA analysis and genetic research. Electromyography of the upper and lower limbs revealed a myopathic pattern. Limb-girdle muscular dystrophy was diagnosed based on the history, proximal muscle weakness, grower’s sign, elevated erythrocyte sedimentation rate, creatine kinase-myocardial binding, creatinine kinase, decreased creatinine (Table 2), and electromyography.

**Table 2:** The laboratory values in the complete blood count report ESR: erythrocyte sedimentation rate; CKMB: creatine kinase-myocardial band; CK: creatinine kinase; mm/hr: millimeters per hour; mg/dl: milligrams per deciliter; U/L: units per liter.

	Patient's observed value	Normal value
ESR	33 mm/hr	0-20 mm/hr
Creatinine	0.4 mg/dl	0. 52-1.04 mg/dl
CKMB	26 U/L	0-16 U/L
CK	343 U/L	30-135 U/L

Investigation

Physiotherapy Intervention

The issues on the patient's list were intricate problems that called for decision-making and action. Fatigue, which can negatively impact a person's quality of life, was the main complaint of the patient. Loss of movement also had to be addressed because it makes it harder to carry out daily duties and keep muscles toned. Moreover, diminished muscle strength makes their physical limitations worse. The patient reported difficulty performing daily activities like combing and using the restroom, which may be related to dynamic balance issues, especially when sitting and standing.

**Figure 1:**
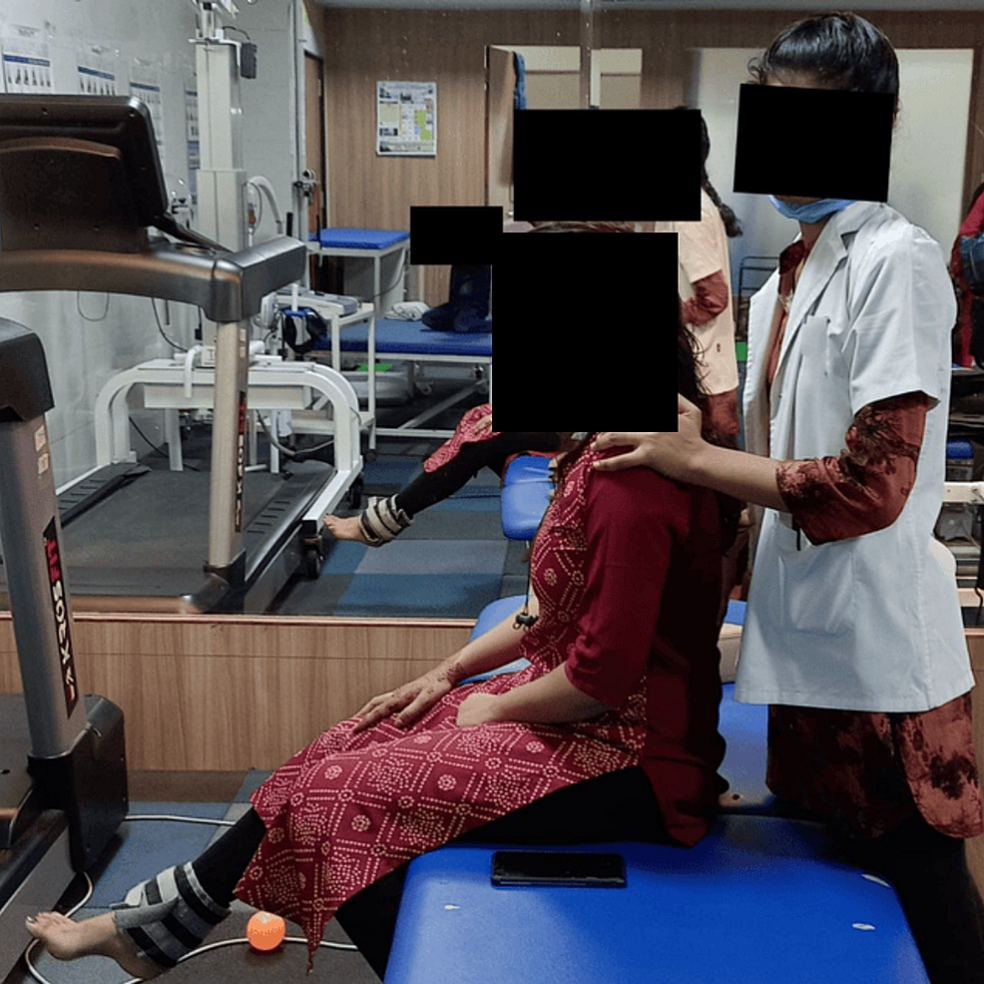
Dynamic quads with a half-kg weight cuff quads: quadriceps; kg: kilogram

**Figure 2:**
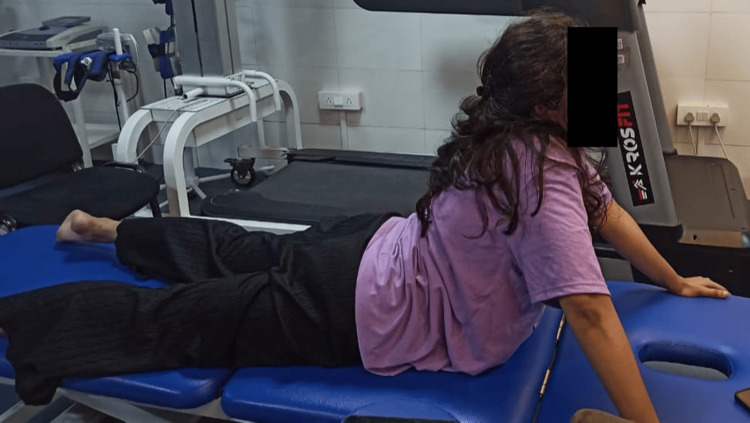
Trunk extension exercises

**Figure 3:**
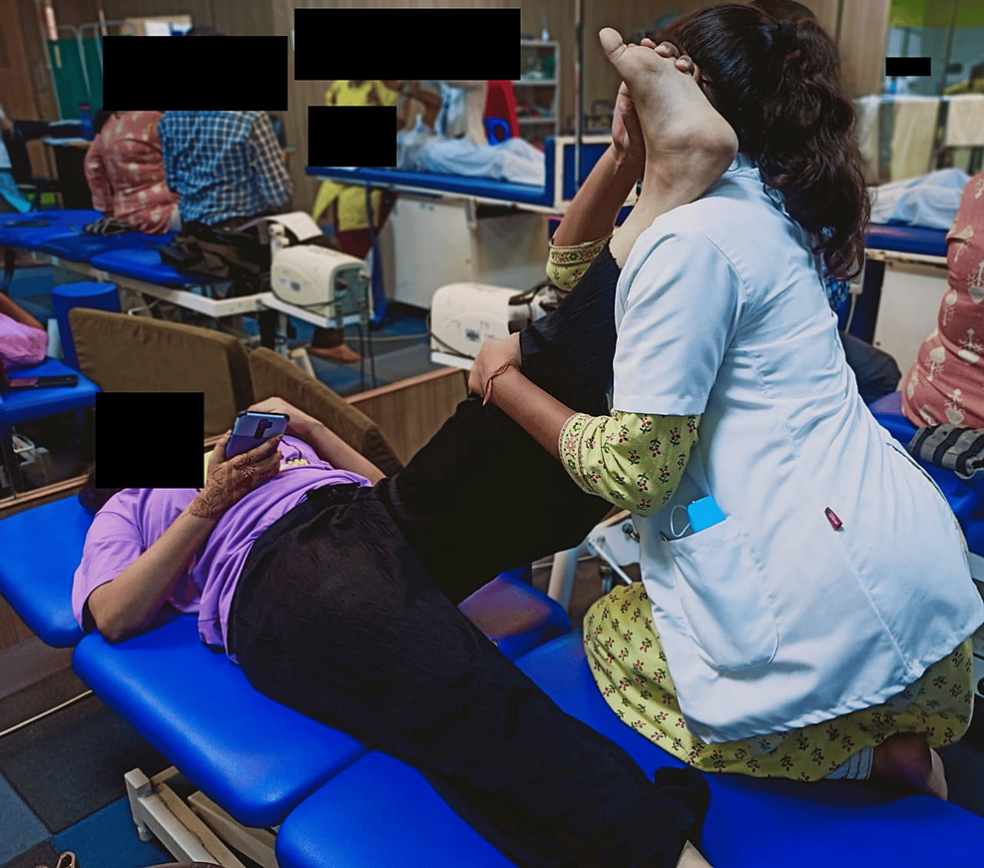
Hamstring stretch

**Figure 4:**
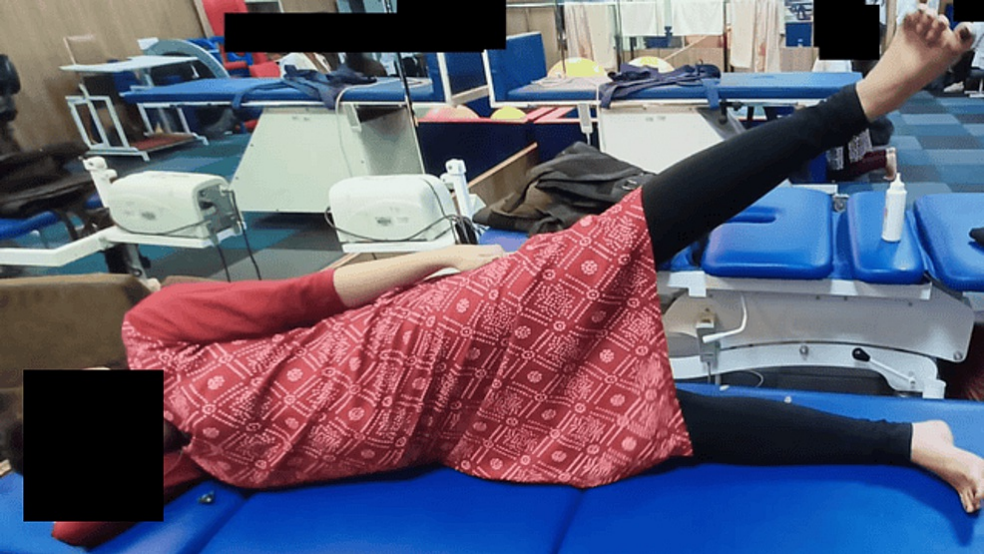
Active range of motion exercises for the lower limbs

Physical therapy's comprehensive and patient-focused goals were intended to deal with these issues. The patient was instructed on techniques to increase muscular strength, improve range of motion, and improve balance and gait. The therapy aimed to improve respiratory health and speed the return to normal activities. This all-encompassing strategy emphasizes the significance of addressing the specific issues and giving back the patient control over their physical health, thereby raising the general quality of life. The management program included physical therapy (Table 3), upper and lower limb mobility exercises with a half-kilogram of weight cuff quadriceps exercises (Figure 1), trunk extension (Figure 2), stretching of the hamstring and Achilles tendon (Figure 3), straight leg raises, and active movement for the lower limb (Figure 4).

**Table 3:** Physiotherapy treatment plan based on the patient's concerns and goals AROM: active range of motion, ADL: activity of daily living, UL: upper limb; LL: lower limb; TA: tendoachilles

Goals	Treatment strategy	Intervention	Progression
Patient Education	Educate the patient and family members about the patient's condition and its effects.	For the patient and his family to be ready to deal with the problem down the road, they were educated about diseases and their effects.	A home exercise program was demonstrated and continued with rehabilitation treatment.
To improve respiratory function	Breathing exercises	Deep breathing exercises (10 repetitions * 2 sets), intercostal stretching (10 repetitions * 2 sets), Jacobson’s relaxation techniques (10 repetitions * 2 sets), Pursed lip breathing (10 repetitions * 4 sets)	Thoracic expansion exercises (10 repetitions * 2 sets) and pursed lip breathing (10 repetitions * 2 sets)
To reduce fatigue	Pacing activity	AROM of scapular elevation, protraction, and retraction (10 repetitions * 2 sets)	Reduce pacing timing
To improve muscle strength and range.	Mobility and strengthening exercises	AROM of scapular elevation, protraction & retraction (10 repetitions * 2 sets)	AROM of scapular elevation, protraction, and retraction. (10 repetitions * 2 sets) with a 5–10 second hold.
		Straight leg raise, dynamic quads (10 repetitions * 2 sets)	Straight leg raise, dynamic quads (10 repetitions * 2 sets) with a 5–10 second hold.
		Static abdominal muscle strengthening (10 repetitions * 2 sets)	Quadruped exercises (10 repetitions * 2 sets)
		Active ROM exercises for both UL and LL with a ½ kg weight cuff (10 repetitions * 1 set)	Active ROM exercises for both UL and LL with a 1 kg weight cuff (10 repetitions * 1 set)
	Stretching exercises	Hamstring and TA stretch (8 repetitions, 10 seconds of hold)	Hamstring and TA stretch (3 repetitions, 30 seconds of hold)
To improve trunk control	Trunk control	Pelvic bridging (10 repetitions * 2 sets), trunk movements: forward, backward, and sideways (10 repetitions * 2 sets)	Wall squats with a Swiss ball (10 repetitions * 2 sets)
To improve balance while sitting	Static and dynamic balance in sitting.	Sit-to-stand exercise (10 repetitions * 1 set)	Pivot transfer in sitting (10 repetitions * 1 set)
To improve balance and gait	Balance and gait training	Tandem standing (5 minutes) and walking (10 repetitions *1 set)	Wobble board training (5 minutes), side-to-side walking, and tandem walking (10 repetitions *1 set)
To improve endurance	Endurance training	Aerobic exercises: cycling, treadmill, or aqua therapy (5–10 minutes a day)	Aerobic exercises: cycling, treadmill, or aqua therapy (15 minutes a day)

Outcome measures

The Berg balance scale (BBS), the multidimensional fatigue scale (MFS), the muscular dystrophy functional rating scale (MDFRS), and the patient-reported outcomes measurement information system (PROMIS-29) were used to evaluate techniques and scales. These scales and practices are critical for tracking the progression of muscular dystrophy, determining how the condition affects a patient's life, and deciding how to prioritize interventions and support services. Patient-rated outcome measures are mentioned (Table 4).

**Table 4:** Outcome measures used to note the progress of the patient MDFRS: muscular dystrophy functional rating scale; PROMIS-29: patient-reported outcomes measurement information system; MFS: multidimensional fatigue scale; BBS: Berg balance scale

Outcome measure scales	Pre-treatment (First day)	Post-treatment (4 weeks)
MDFRS	75/96	83/96
PROMIS-29	Physical function: 8/20; anxiety: 12/20; depression: 12/20; fatigue: 16/20; sleep disturbance: 4/20; ability to perform activity: 16/20; pain interference: 4/20 pain intensity: 0/10	Physical function: 14/20; anxiety: 8/20; depression: 10/20; fatigue: 12/20; sleep disturbance: 4/20; ability to perform activity: 16/20; pain interference: 4/20; pain intensity: 0/10
MFS	60/100	40/100
BBS	48/56	52/56

Follow-up

Limb-girdle muscular dystrophy is a progressive disorder. Thus, regular follow-up consultations with the physiotherapist are needed to determine changes in the patient's health and alter the treatment plan accordingly. After four weeks of therapy, the patient could do activities of daily living and needed to develop muscle strength, range of motion, balance, gait and postural stability, respiratory function, and functional training. Her motivation came from a combination of her own experiences, success, continued coaching, and dedication to overall wellness-which has a significant influence on their growth and well-being and willingness to participate in the suggested program.

## Discussion

Patients with LGMD have a gradual deterioration of functioning skeletal muscles and progressive weakness. Based on the pathophysiology, level of clinical involvement, and rate of progression, LGMD-related impairment is determined [8]. The BBS, the MDS, the MDFRS, and the PROMIS-29 are used to evaluate techniques and scales that are crucial for tracking the progression of muscular dystrophy, determining how the condition affects a patient's life, and deciding how to prioritize interventions and support services. Medical personnel typically combine these techniques with clinical assessments and other methods like investigations, therapies, surgery, and medications to provide complete care to patients with muscular dystrophy [15-17]. Weakness, fatigue, poor endurance, and related functional deficiencies are caused in these patients by the loss of active muscle fiber and atrophy brought on by inactivity.

There is currently no effective pharmacologic therapy for LGMD, even though numerous therapeutic regimens have been proposed [18]. Patients must, therefore, rely on symptomatic care, in which ongoing physical therapy is essential. Maintaining strength, functional capacity, and a way of life is the major goal of physiotherapy. Exercise is crucial to preserve and enhance strength, endurance, gait, and way of life in LGMD patients [13]. Strength training (progressive resistance exercise) improves physical performance by increasing muscle mass, lean body mass, contractile force, and power. Exercise promotes muscle growth by boosting muscle proteins, especially actin and myosin, and myofibril deoxyribonucleic acid content [19]. The ability to sustain sub-maximal activity for longer periods while exerting less effort demonstrates how endurance training lessens fatigue and results in physiological adaptations distinct from those brought on by strength training [4, 10]. Strength and endurance training appeared to be a reliable and effective method during the first recovery of LGMD [20]. When studying patients with progressive illnesses like LGMD, treatment planning is significantly more important, especially when those patients undergo a rapid loss of health [9].

## Conclusions

Limb-girdle muscular dystrophy was diagnosed based on the history of weakness in the proximal muscle, Gower’s sign, elevated erythrocyte sedimentation rate, increased creatine kinase-myocardial binding, raised creatinine kinase, decreased creatinine, and electromyography. Muscular dystrophy was discovered through a muscle biopsy. Significant differences were seen in the various outcome variables between the baseline evaluation and after four weeks of physiotherapy treatment, and the recommendations for exercise used in the case report primarily emphasize the empirical benefits of strength and endurance training. The therapy has significantly impacted the patient's functional skills based on improved scores on scales like the MDFRS, BBS, MFS, and PROMIS-29. Exercises with a low-to-moderate level of intensity may support the maintenance of muscle function, increase joint mobility, and improve general physical health.
